# CRISPR/Cas9-Mediated Gene Correction to Understand ALS

**DOI:** 10.3390/ijms21113801

**Published:** 2020-05-27

**Authors:** Yeomin Yun, Yoon Ha

**Affiliations:** 1 Department of Neurosurgery, Spine and Spinal Cord Institute, College of Medicine, Yonsei University, Seoul 03722, Korea; ymyun@yuhs.ac; 2 Brain Korea 21 PLUS Project for Medical Science, College of Medicine, Yonsei University, Seoul 03722, Korea

**Keywords:** amyotrophic lateral sclerosis (ALS), CRISPR/Cas9, induced pluripotent stem cells (iPSCs), gene correction

## Abstract

Amyotrophic lateral sclerosis (ALS) is a neurodegenerative disease caused by the death of motor neurons in the spinal cord and brainstem. ALS has a diverse genetic origin; at least 20 genes have been shown to be related to ALS. Most familial and sporadic cases of ALS are caused by variants of the *SOD1*, *C9orf72,* *FUS*, and *TARDBP* genes. Genome editing using clustered regularly interspaced short palindromic repeats/CRISPR-associated system 9 (CRISPR/Cas9) can provide insights into the underlying genetics and pathophysiology of ALS. By correcting common mutations associated with ALS in animal models and patient-derived induced pluripotent stem cells (iPSCs), CRISPR/Cas9 has been used to verify the effects of ALS-associated mutations and observe phenotype differences between patient-derived and gene-corrected iPSCs. This technology has also been used to create mutations to investigate the pathophysiology of ALS. Here, we review recent studies that have used CRISPR/Cas9 to understand the genetic underpinnings of ALS.

## 1. Introduction

Amyotrophic lateral sclerosis (ALS) is the most common form of motor neuron disease (MND). The term “amyotrophic” derives from “a”, which means no; “myo”, which means muscle; and “trophic”, which refers to nourishment. Furthermore, “lateral” refers to the lateral corticospinal tracts and “sclerosis” refers to scarring of lateral region [1]. Taken together, “amyotrophic lateral sclerosis” denotes a lack of nourishment that leads to muscle wasting in the spinal cord, where descending motor pathways carry motor signals from the brain down to the target muscle or organ. ALS, which is diagnosed using an electromyography (EMG) test, is characterized by the loss of motor neurons and muscle weakness, which results in respiratory failure and death within 3–5 years of onset [2].

A study in 1993 showed that the *SOD1* gene was associated with some cases of ALS [3]. Since this seminal study, the genetic etiology of ALS has expanded dramatically to include more than 20 different genes (ALSoDs) [4]. The onset of ALS usually occurs in adulthood, but juvenile-onset ALS has been observed in patients with mutations in the *FUS* gene [5]. There is no definitive cure for ALS, although Riluzole increases patient survival by three months; the United States Food and Drug Administration (FDA) has also recently approved a new treatment, Radicut [6,7,8,9].

Clustered regularly interspaced short palindromic repeats/CRISPR-associated system 9 (CRISPR/Cas9) genome-editing technology has advanced the field of genome engineering [10]. CRISPR/Cas9 has been adapted from a natural genome-editing system of bacteria. In the bacterial immune system, bacteriophages recognize foreign genetic elements that occur as a result of infection and destroy these foreign DNA fragments. The CRISPR/Cas9 system works similar to cut and replace DNA fragments. Although other genome editing tools exist, such as zinc finger nucleases (ZFNs) and transcription activator-like effector nucleases (TALENs), CRISPR/Cas9 is simple and inexpensive to use compared with prior tools [11,12]. In this review, we focus on recent studies that have used CRISPR/Cas9-mediated gene correction and disease modeling to study ALS. In particular, we briefly summarize and discuss prominent mutations in the *SOD1*, *C9orf72, FUS*, and *TARDBP* genes and their roles in ALS, as revealed by CRISPR/Cas9 technology.

## 2. Amyotrophic Lateral Sclerosis (ALS)

ALS, also known as Lou Gehrig’s disease, is the most common MND. ALS is characterized by muscle weakness and the loss of motor neurons in the spinal cord and brainstem. Clinical symptoms are anatomically divided into two categories based on whether symptoms start in the limbs or bulbar region. Limb onset displays symptoms in the upper (arm or hand) or lower limbs (leg or foot), and bulbar onset patients have symptoms including weakness of muscle controls in speech and swallowing [13,14,15]. The age of onset is typically 55–60 years old, and the worldwide prevalence is approximately 1.68 per 100,000 individuals [16,17]. One study estimates that the proportion of individuals affected by ALS will increase by 69% by 2040, primarily owing to a rapidly aging population [18]. The average survival of patients with ALS is 2–5 years. Approximately 20% of patients survive for five years, eventually succumbing to respiratory failure, whereas 5% of patients may live for 20 years [19,20].

**Figure 1:**
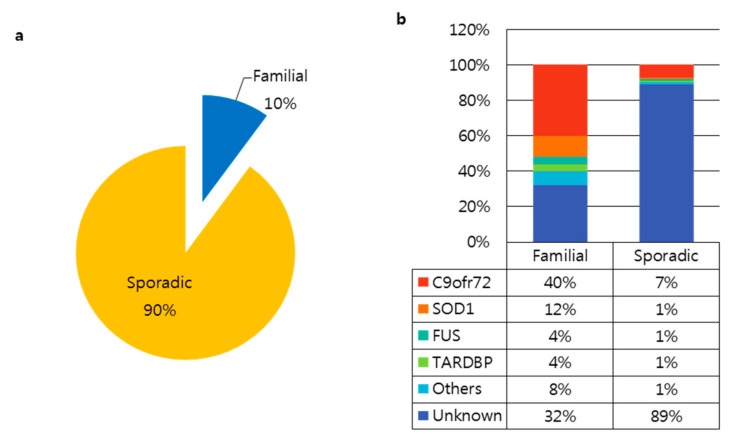
Genetic causes of amyotrophic lateral sclerosis (ALS). (**a**) Prevalence of familial and sporadic ALS cases. (**b**) Proportion of causative genes in familial and sporadic ALS [25].

There are two types of ALS, familial (fALS, 10% of cases) and sporadic (sALS, 90% of cases) (Figure 1a). Since the discovery of superoxide dismutase 1 (*SOD1*) in 1993, over 20 genes have been linked to ALS. According to the Amyotrophic Lateral Sclerosis online Database (ALSoD), over 100 genes may be linked to ALS [3,21,22]. Of these, the most common genetic causes of ALS involve mutations in the *SOD1*, *C9orf72, FUS*, and *TARDBP* genes (Figure 1b). The Genome Aggregation Database (gnomAD) provides whole-genome or exome sequences of various diseases. We explored variants in *SOD1*, *C9orf72, FUS*, and *TARDBP* in the gnomAD v2.1.1 database as well as the Human Genome Variation Society (HGVS) database from the National Institutes of Health (NIH) to gather more information about each variant, including protein changes and dbSNPs (Table 1). Gene variants are classified into five categories according to their clinical significance: pathogenic, likely pathogenic, uncertain significance, likely benign, and benign [23]. There are 36 ALS-related variants in SOD1, 11 variants in *FUS,* and 14 variants in *TARDBP* with a pathogenic clinical significance. Variants in *SOD1*, *C9orf72, FUS*, and *TARDBP* account for up to 60% of familial ALS and 10% of sporadic ALS; the cause of the remaining cases remains unclear. The most common genetic cause of ALS is the (G_4_C_2_)n repeat expansion in *C9orf72* [2,24].

A recent study, Project MinE, has begun to analyze the whole-genome sequence data of at least 15,000 patients with ALS worldwide [26]. Whole-genome sequencing is a promising tool to discover unknown genetic variants that contribute to disease. In a pilot analysis of 1169 patients with ALS, several rare genetic variations were found [27]. Several mutations were genetically associated with cellular pathology, which is characterized by motor neuron degeneration due to an interplay of axonal dysfunction, oxidative stress, mitochondrial dysfunction, microglia activation, and protein aggregation [13,28].

## 3. Targeted Genome Editing Using CRISPR/Cas9

CRISPR (clustered regularly interspaced short palindromic repeats)/CRISPR-associated system (Cas) is an immune system derived from prokaryotes that can be used to target and edit specific sequences. CRISPR sequences are located in prokaryotes such as bacteria and archaea. New foreign DNA fragments derived from infection are integrated into host CRISPR sequences and transcribed into CRISPR RNA. These RNA sequences are used as a guide to recognize foreign genetic elements derived from previously infected prokaryotes. The Cas protein is an enzyme that cleaves DNA fragments to match the CRISPR RNA [29,30].

To use CRISPR/Cas, a researcher designs small guide RNA (sgRNA) near the proto-spacer adjacent motif (PAM) sequences for Cas nuclease to bind target DNA sequences recognized by sgRNA. The Cas protein-coding gene and sgRNA plasmids are usually transfected via electroporation or lipid-based transfection agents. Other CRISPR/Cas9 delivery methods include ribonucleoprotein (RNP) complex and packaging into lentivirus or adeno-associated virus (AAV). Once in the nucleus, Cas nuclease binding leads to a double-strand break (DSB) in DNA that is recovered by the cell’s own repair system. These DSBs are commonly repaired by non-homologous end joining (NHEJ) or homology-directed repair (HDR) in the presence of a donor template. The difference between NHEJ and HDR is that the NHEJ mechanism generates insertions and deletions (indel) at a cleavage site, resulting in the knockout of a protein coding gene, whereas the HDR mechanism induces insertions or replaces DNA fragments with a donor template [31,32].

Cas9 from *Streptococcus pyogenes* (SpCas9) is widely used for the CRISPR/Cas system. Various other subtypes of the Cas protein, including saCas9, c2c2, RCas, and dCas, have also been identified and developed for applications in genome editing [33]. Each Cas protein has a unique PAM sequence and different protein sizes for different applications. These proteins have specific cleavage targets in DNA and RNA, and generate single-strand nicks (i.e., Cas9n) that activate or inhibit transcription (i.e., CRISPRa or CRISPRi) [34,35].

**Figure 2:**
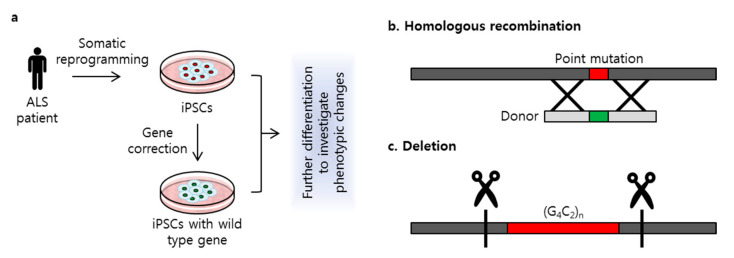
Strategy of clustered regularly interspaced short palindromic repeats/CRISPR-associated system 9 (CRISPR/Cas9)-mediated gene correction. (**a**) Patient-derived induced pluripotent stem cells (iPSCs) were used to perform gene correction. Isogenic cell line was generated to investigate phenotypic changes comparing iPSCs with genetic mutation; (**b**) A point mutation was target specific corrected using a donor via homology-directed repair (HDR) mechanism; (**c**) Repeat expansion in *C9orf72* was deleted with two sgRNAs.

Genome editing using CRISPR/Cas9 is used in disease research to study genetic modifications in various cell types. Many years ago, mutagenesis or mutant transfection was widely used to investigate the genetic effects of mutations on disease [31,36]. Since the development of reprogramming technology, somatic cells have been used for induced pluripotent stem cell (iPSC) generation [37]. These iPSCs have the potential to differentiate into various cell types, such as neurons, liver cells, myoblasts, and cardiomyocytes. Patient-derived iPSCs provide an unlimited source of cells with which to study and treat disease [38,39,40,41]. For genetic disorders in particular, pathologic effects of mutations can be investigated in iPSCs using CRISPR/Cas9 to correct mutations (Figure 2a). The correction of specific mutations related to ALS can thus provide important insights into both verifying the pathologic effects of these mutations and identifying targets for therapeutic interventions.

In both fALS and sALS, the majority of causative genetic mutations are found in the *SOD1, C9orf72*, *FUS*, and *TARDBP* genes. Most pathogenic variants are the result of missense mutations, although repeat expansion in the intron region of the *C9orf72* gene has been identified as a known cause of ALS. Patient-derived iPSCs are an important disease model to study ALS. One drawback to patient-derived iPSCs, however, is the phenotypic variation, which makes it difficult to determine which genetic mutations are relevant to ALS. Here, we review recent studies that have applied CRISPR/Cas9 to patient-derived iPSCs to study genetic mutations in ALS. These methods have included the generation of isogenic cell lines with the same genetic background as the iPSCs to understand the genetic etiology and pathophysiology of ALS (Table 2).

### 3.1. Superoxide Dismutase 1 (SOD1)

Mutant *SOD1* was the first gene to be linked to ALS. *SOD1* is located on chromosome 21q22.1 and contains five exons. More than 100 *SOD1* mutations have been identified, 30 of which are associated with ALS, according to the genomAD and ClinVar online databases [42]. Mutations in *SOD1* are reported in ~12% of fALS and ~1% of sALS cases [25,43]. *SOD1* is an antioxidant enzyme that binds to copper and zinc to break down superoxide radicals, preventing cell damage. Mutations in *SOD1* lead to neuronal excitability, endoplasmic reticulum stress, mitochondrial dysfunction, oxidative stress, impaired protein transport, accumulation of misfolded *SOD1* protein, and inflammation, all of which may increase cell death [44,45].

A recent study [46] conducted a targeted gene correction of the SOD1 A272C mutation in patient-derived iPSCs. Fibroblasts harboring the A272C mutation were obtained from a patient with ALS and reprogrammed to generate iPSCs. Guide RNA was designed to include a PAM sequence that targeted the A272C mutation. A donor template to replace this point mutation was co-transfected with Cas9 nuclease and sgRNA via electroporation (Figure 2b). Because ALS is an MND, patient-derived iPSCs and the gene-corrected isogenic cell line were differentiated into motor neurons to compare neuronal differentiation capacity. RNA sequencing revealed significantly altered gene expression between the patient-derived motor neurons and the gene-corrected isogenic motor neurons. Gene ontology analysis revealed that expression was altered in RNA related to nervous system activity, signal transduction, and endoplasmic reticulum homeostasis. A similar study [47] applied this CRISPR/Cas9 gene-correction strategy to the SOD1 E100G mutation. Targeted gene correction was performed in iPSCs derived from a patient with the E100G mutation, which then were differentiated into motor neurons. Correction of the SOD1 E100G mutation in an isogenic cell line increased motor neuron proportion, soma size, and neurite length and decreased cell death. Transcriptional changes detected by RNA sequencing identified several up- or down-regulated pathways, including those related to AP1, oxidative phosphorylation, and ion channel transport. The functional gene network showed that these pathways share functional interactions and cascades. On the basis of these results, the authors hypothesized that activated ERK and JNK signaling is critical to neurodegeneration in mutant SOD1 motor neurons. Treatment with small-molecule inhibitors that target ERK, MAPK, JNK, WNT, TP53, and CDK kinases decreased motor neuron degeneration, suggesting that these pathways are critical in SOD1-linked ALS. A study by Imamura et al. [48] used CRISPR/Cas9-mediated gene corrected cell lines in drug screening assay as a control. iPSCs were generated from one ALS patient carrying the L144FVX mutation in the SOD1 gene and mutation was corrected by inserting wild type SOD1 sequences with homologous arms. The corrected cell line showed reversal of SOD1-associated pathologic phenotype, including increased motor neurons survival, degradation of misfolded SOD1 protein, and reserved internal ATP level. On the other hand, high-throughput screening discovered that more than half of hit targeted the Src/c-Abl signaling pathway in motor neurons differentiated from iPSCs. The treatment of bosutinib, one of the directly inhibited Src/c-Abl, showed therapeutic effects on motor neurons’ survival, decreased phosphorylation of Src/c-Abl, promoted autophagy activity, adecreased misfolded mutant SOD1, and rescue of ATP, as did gene correction of L144FVX. In this study, the authors revealed the potential application of the gene corrected isogenic cell line used as a control to validate the therapeutic effects of drugs on ALS disease.

### 3.2. C9orf72

The hexanucleotide (G_4_C_2_) repeat expansion in *C9orf72* is the most common genetic cause of ALS and is also common in frontotemporal dementia (FTD). This repeat expansion is responsible for up to 40% of fALS and 7% of sALS cases [25,43,49]. The repeat is located in intron1 of the C9orf72 gene. The number of repeats is highly variable within an individual, ranging from hundreds to thousands of repeats; less than 30 repeats is considered normal [50]. Mutant *C9orf72* has several pathologic neurodegenerative phenotypes, including reduced protein expression levels, promoter hypermethylation, excitotoxicity, RNA foci formation, and generation of dipeptide repeats (DPRs). Transcription of this repeat expansion produces RNA with abnormal secondary structures that aggregate to form intranuclear foci, which results in the disruption of transcription and cytoplasmic transport [51,52]. Repeat-associated non-AUG (RAN) translation produces toxic DPR proteins [53]. Poly GR(glycine arginine), one of the dipeptide chains, is accumulated in patients with *C9orf72*-associated ALS and FTD [54,55].

Mutant *C9orf72* genes with 800–1050 repeats from patients with ALS are also associated with RNA foci formation and hypermethylation [56]. CRISPR/Cas9 genome editing technology with two sgRNAs has been designed to excise repeat expansions via the cleavage and removal of the repeat region (Figure 2c). Deletion of the repeat expansion results in decreased RNA foci formation and reduces methylation in repeat knockout iPSC-derived neurons. In contrast, deletion of the normal allele did not reverse the methylation level, which indicates that the hexanucleotide (G_4_C_2_) expansion is the cause of hypermethylation within the GC-rich region of *C9orf72*. Another study that used iPSC lines with up to 638, 760, and 960 repeats in *C9orf72* showed that the *C9orf72* mutation increased AMPA receptor expression, leading to enhanced excitotoxicity via intracellular Ca^2+^ overload [57], as well as RNA foci formation and DPR toxicity, but no differences in neuron excitability in iPSC-derived motor neurons were observed. A genome-editing approach that deleted the repeat expansion was able to rescue the AMPA-induced excitotoxicity and reduced the RNA foci formation and DPR expression. RNA sequencing also showed that deletion of the repeat expansion altered the gene expression in genes related to RNA processing, protein targeting, nuclear transport, synaptic transmission, and the regulation of phosphate metabolism [58].

Unlike the *hSOD1* G93A transgenic mouse model, deletion of *C9orf72* in the mouse does not produce an ALS-like phenotype [59]. As an alternative, *Drosophila* expressing 58 repeats of G_4_C_2_ or poly GR overexpression has been used to investigate the involvement of the Ku80 protein in ALS neurodegeneration [60,61]. Studies have confirmed that Ku80 protein levels are increased in both this *Drosophila* model and in C9orf72 iPSC-derived motor neurons with 70, 590, 1000, or 1100 repeats in each cell line. Ku80 is an essential DNA repair protein. The elevated expression of Ku80 leads to an increase in downstream pro-apoptotic proteins such as PUMA, BAX, and cleaved caspase-3. The application of genome editing using two sgRNAs reduced Ku80 levels and prevented nuclear RNA foci formation in iPSC-derived motor neurons with the corrected repeat expansion. Using CRISPR/Cas9, one copy of Ku80 in the *C9orf72* iPSC was deleted, which resulted in the reduction of Ku80 expression as well as PUMA and cleaved caspase-3 levels. These studies, therefore, demonstrate that the CRISPR/Cas9 approach can be applied to the *C9orf72* repeat expansion to validate pathologic phenotypes in patient-derived iPSCs and identify gene-based therapeutic approaches.

### 3.3. Fused in Sarcoma (FUS)

The fused in sarcoma (*FUS*) gene encodes an RNA-binding protein that is located primarily in the nucleus and composed of seven domains: QGSY-rich, Gly-rich, RNA recognition motif (RRM), Arg-Gly-Gly1 (RGG1), zinc finger (ZnF), RGG2, and nuclear localization signal (NLS) [62]. The majority of disease-causing *FUS* mutations are heterozygous with an autosomal-dominant inheritance pattern [63]. In addition, most *FUS* mutations affect the C-terminal nuclear localization signal. The known functions of *FUS* include DNA damage repair, transcription, and splicing [64]. The effect of mutant *FUS* in neurodegeneration and other biological processes is unknown; however, one hallmark of mutant *FUS* is cytoplasmic mislocalization, which may be related to a toxic gain of function in the cytoplasm [65]. Previous findings have suggested that mutant *FUS* may impact DNA repair, energy metabolism, and axonal transport. Recent studies have used CRISPR/Cas9 gene editing to study the impact of mutant *FUS* on ALS pathologic phenotypes in patient-derived iPSCs. A study by Wang et al. [46] first demonstrated CRISPR/Cas9-mediated gene correction of the *FUS* G1566A missense mutation in ALS patient-derived iPSCs. Another study used the CRISPR/Cas9 system to correct the recessive *FUS* H517Q mutation to the wild-type sequence in patient-derived iPSCs. Both mutant and corrected iPSCs were differentiated into Isl1/Tuj1-positive motor neurons to investigate the expression of the MAPK family. These results showed that both p38 and ERK kinase are activated in mutant *FUS*, indicating that the activation of MAPK signaling is a key pathway in *FUS*-mediated neurodegeneration in ALS [47]. Motor neurons derived from patients with ALS with the *FUS* mutation typically show cytoplasmic mislocalization [64]. Motor neurons carrying the *FUS* P525L and R521H mutations also exhibit mislocalization of *FUS*, compared with no aggregation in healthy control cells.

Electrophysiological changes and defects in axonal transport have also been observed in motor neurons with mutant *FUS*. The movement of endoplasmic reticulum vesicles is also significantly reduced, which results in impaired axonal transport [66]. To confirm whether these observed pathologic phenotypes were the result of the *FUS* mutation, CRISPR/Cas9-mediated gene correction was applied to iPSCs derived from patients with the *FUS* R521H mutation. Isogenic cell lines with the same genetic background as the mutant iPSCs were also established. Mitochondrial transport experiments showed that gene correction rescued the number of total and moving mitochondria as well as cytoplasmic *FUS* localization. Moreover, these experiments found that an HDAC6 inhibitor is an effective drug for targeting axonal transport impairment. This study thus demonstrates that gene correction can mediate the investigation of *FUS*-related disease pathologic phenotypes and may have the potential to be used for therapeutic discovery and validation.

Oxidative stress promotes genome damage and neurodegeneration results in motor neuron death, which is consistent with increased markers of oxidative stress in patients with ALS [67,68]. In healthy neurons, *FUS* is localized predominantly to the nucleus and promotes genome repair by recruiting PARP-1 dependent XRCC1/DNA ligase IIIα (LigIII) in response to DNA damage response (DDR) signaling [69]. ALS patient-derived motor neurons with *FUS* P525L and R521H mutations exhibit an approximately threefold increase in the cytoplasmic accumulation of *FUS* as well as a cytotoxic response to H_2_O_2_ and >50% decreased efficiency of nick ligation owing to the delay of DNA break repair [70]. Gene-corrected isogenic cell lines have been established using CRISPR/Cas9 to investigate the direct attribution of *FUS* mutations to DNA ligation impairment. Correction of these *FUS* mutations rescued DNA ligation defects, as assessed by ligation assay, and DNA integrity was also restored, indicating that mutant *FUS* fails to correct DNA ligation defects caused by oxidative stress. This study suggests that LigIII function may be a therapeutic target in patients with *FUS* mutations.

Energy metabolism is a critical component in neurodegeneration as well as genome repair, because most biological processes, including neuronal firing and axonal transport, are energy demanding [71,72]. The elevation of glycogen stores observed in patients with ALS implies a defect in mitochondrial function and a failure in glucose matabolism, which has been hypothesized to cause the hallmark motor neuron degeneration in ALS. Mislocalization caused by mutant *FUS* increased enzymatic interactions in glucose metabolism [73], indicating that energy metabolism may be involved in *FUS*-related ALS degeneration. Substrate-tracing experiments using radiolabeled ^13^C_6_—glucose and ^13^C_3_—lactate reveal the involvement of oxidative metabolism, but not the glycolytic pathway in motor neurons differentiated from iPSCs. In motor neurons carrying an *FUS* mutation, however, there was no evidence of altered mitochondrial morphology, such as intermembrane space and cristae patterns, as measured by transmission electron microscopy (TEM), whereas *FUS* overexpression was associated with a defect in the mitochondrial structure. Radioactive tracer analysis using ^13^C was performed to investigate *FUS*-related changes in glucose and/or lactate metabolism in *FUS*-mutant versus gene-corrected motor neurons. Although lactate was revealed as a main energy source in motor neurons, there was no altered contribution of glucose or lactate to glycolytic or TCA metabolites after gene correction, indicating that there is no metabolic dysfunction in *FUS*-related ALS phenotypes [5]. The use of CRISPR/Cas9 genome editing in patient-derived iPSCs also reveals that mutant *FUS* does not alter metabolic pathways, providing further evidence that metabolic dysfunction is not involved in *FUS*-related ALS.

### 3.4. TAR-Binding Protein Gene (TARDBP)

The *TARDBP* gene is located on chromosome 1p36.22 and contains six exons encoding TAR-DNA binding protein TDP-43. It is a DNA/RNA binding protein and functions in a variety of RNA metabolism such as splicing, stability, and trafficking. Mutations in *TARDBP* gene were first reported by Sreedharan et al. in 2008 [74]. Since then, many mutations have been identified through genetic analysis in ALS patients, and most mutations are conserved within exon 6 of the *TARDBP* gene, which encodes the carboxy-terminal glycine-rich domain [75]. TDP-43 protein undergoes post-translational modification including ubiquitination, phosphorylation, acetylation, cysteine oxidation, and PARylation. Ubiquitinated and hyper-phosphorylated TDP-43 forms cytoplasmic inclusion bodies—a pathological hallmark of ALS. Mutations in the C-terminal region hasten aggregations, mislocalization to the cytoplasm, and cytotoxicity [76].

In the nervous system, brain-derived neurotrophic factor (BDNF) is essential for neuron survival, differentiation, and synaptic plasticity. It is produced and regulated to the secretory pathway in neurons by binding of Sortilin (encoded by *sort1* gene) to the pro-domain of BDNF. TDP-43 regulates the splicing of mRNA encoding Sortilin. Loss of TDP-43 leads to the generation of isoform owing to additional exon 17b and soluble Sortilin protein. Therefore, a study by Tann et al. hypothesized that TDP-43 dysfunction caused by mutations in *TARDBP* gene altered BDNF secretion and synaptic plasticity [77]. In a mouse model, the authors confirmed that hippocampal CA1-specific knockout of TDP-43 increased exon 17b mRNA of *sort1* mRNA and reduced dendrite complexity and spine. To investigate the effect mutations could have in human neurons, CRISPR/Cas9 was used to correct M337V in three iPSCs lines from a patient. Corrected iPSCs were differentiated into excitatory cortico/hippocampal-like neurons. All isogenic lines showed a similar mRNA level of *TARDBP* and *SORT1*, but a decreased level of exon 17b mRNA of *sort1* compared with cells bearing M337V. Lentivirus expressing human pro-BDNF was transduced to neurons and to assess BDNF secretion. M337V displayed a decreased level of BDNF, and significantly increased in all isogenic gene corrected neurons. This study indicated that TDP-43 mutation is associated to Sortilin isoform and BDNF secretion, and the gene correction strategy demonstrated that M337V mutation in *TARDBP* is a direct cause of abnormal BDNF secretion.

## 4. CRISPR/Cas9-Mediated In Vivo Therapeutic Approach

A transgenic mouse model that overexpresses the human *SOD1* G93A mutation is widely used in studies of ALS. This mouse model expresses a similar pathologic phenotype to human ALS, including short survival and loss of motor behavior function. Recent studies using this mouse model [78,79] have reported that CRISPR/Cas9 disturbs the expression of mutant *SOD1*, which may be a promising potential therapeutic strategy. These studies have hypothesized that the reduced expression of mutant protein reverses the disease phenotype.

**Table 2:** Studies of clustered regularly interspaced short palindromic repeats/CRISPR-associated system 9 (CRISPR/Cas9)-mediated genome editing in amyotrophic lateral sclerosis. N/R, not recorded; ALS, amyotrophic lateral sclerosis; iPSC, induced pluripotent stem cell; FTD, frontotemporal dementia.

Study	Gene	Variants	Model	Gene Editing Efficiency
**Homologous recombination**				
Wang et al., 2017 [46]	SOD1	A272C	Human ALS patient-derived iPSCs	20%
Bhinge et al., 2017 [47]	SOD1	E100G	Human ALS patient-derived iPSCs	0.5% (one of ~ 180 colonies)
Imamura et al., 2017 [48]	SOD1	L144FVX	Human ALS patient-derived iPSCs	Selected with puromycin
Guo et al., 2017 [66]	FUS	R521H	Human ALS patient-derived iPSCs	Selected with puromycin
Wang et al., 2017 [46]	FUS	G1566A	Human ALS patient-derived iPSCs	1%
Bhinge et al., 2017 [47]	FUS	H517Q	Human ALS patient-derived iPSCs	0.5% (one of ~ 180 colonies)
Wang et al., 2018 [70]	FUS	R521H, P525L	Human ALS patient-derived iPSCs	Selected with puromycin
Vandoorne et al., 2019 [5]	FUS	R521H, P525L	Human ALS patient-derived iPSCs	Selected with puromycin
Tann et al., 2019 [77]	TARDBP	M337V	Human ALS patient-derived iPSCs	FACS sorting
**Deletion**				
Pribadi et al., 2016 [56]	C9orf72	GGGGCC	Human FTD/ALS patient-derived iPSCs	11.1% (66 of 593 colonies)
Selvaraj et al., 2018 [58]	C9orf72	GGGGCC	Human ALS patient-derived iPSCs	0.6% ~ 4.5%
Lopez-Gonzalez et al., 2019 [61]	C9orf72	GGGGCC	*C9orf72* patient-derived iPSCs	N/R
Duan et al., 2019 [79]	SOD1	G93A	*hSOD1* G93A transgenic mouse	N/R
**Indel formation**				
Gaj et al., 2017 [78]	SOD1	G93A	*hSOD1* G93A transgenic mouse	N/R

Several sgRNAs that do not overlap with the G93A mutation have been designed for application to other *SOD1* mutations. For in vivo genome editing such as that using animal models, AAV is the safest delivery vehicle. Because AAV has a limited capacity to harbor the plasmid, however, Cas9 nuclease from *Staphylococcus aureus* (SaCas9) is often used, thanks to its smaller size than spCas9 [80]. The plasmid, consisting of SaCas9 under the CMV promoter and sgRNA under the U6 promoter, is packaged into AAV. In a study of the *hSOD1* G93A mouse model, this AAV was injected via the facial vein at postnatal day 0–1. Four weeks after injection, Western blot and histological analysis confirmed reduced expression of the SOD1 protein and an increased number of motor neurons in the lumbar spinal cord. Furthermore, these findings showed that genome editing improved motor function, reduced muscle atrophy, delayed disease onset, and increased survival compared with a control model.

Taken together, studies using CRISPR/Cas9-mediated in vivo genome editing of missense mutations in *SOD1* show that this technique can be used not only to identify the underlying molecular mechanism but also to reverse the pathologic phenotype in patient-derived disease models, and thus may play a potential role in gene therapy for ALS.

## 5. Conclusions and Future

In this review, we discussed recent studies that have used CRISPR/Cas9-mediated gene correction to investigate the pathophysiology of ALS using patient-derived iPSCs. Although CRISPR/Cas9 technology is simple and easy to use across several research applications, there remain limitations with this approach. One critical concern is off-target effects, which could occur if the specificities of Cas9 and sgRNA are reduced such that they recognize and cleave non-target DNA sites [81]. Another limitation is the low (<1%) efficiency of gene correction through the HDR mechanism. Techniques such as timed delivery with cell cycle synchronization [82,83] and suppression of NHEJ key molecules [84] are needed to enhance HDR genome engineering rates.

As mutant SOD1 was revealed as a genetic cause of ALS in 1993, over 20 genes associated with ALS have been identified; however, more than 80% of patients with ALS do not exhibit known genetic variants [85]. Clinical differences between patients may imply that multiple mechanisms are involved with various genetic causes. According to ALSoD, over 100 genes are listed as ALS-associated genes. Recently, Vildan et al. analyzed *SOD1*, *C9orf72*, *FUS*, *TARDBP,* and *UBQLN2* to find common genetic alterations in 30 patients with ALS [86]. In addition, many European countries and the United States have joined Project MinE to analyze the genes of 15,000 patients with ALS and 7500 controls. The aim of this project is to better understand the genetic background of ALS. Online open access resources also exist to obtain information about gene variants in various diseases, including ClinVar and Miner. The application of genetic information, advanced methods in genome engineering with CRISPR/Cas9, and in vitro disease modeling using iPSCs is likely to facilitate the discovery of disease-causing mutations and further investigate the underlying pathophysiology of various diseases. It is the hope that this strategy will ultimately lead to a thorough investigation of the various mechanisms underlying ALS to identify treatments and a cure for ALS.

## Figures and Tables

**Table 1:** Amyotrophic lateral sclerosis (ALS)-associated pathogenic variants in *SOD1*, *C9orf72, FUS,* and *TARDBP* (Genome Aggregation Database (gnomAD) v2.1.1).

Nucleotide	Protein	dbSNP	Mutation Type
**SOD1**			
NM_000454.4:c.14C >T	NP_000445.1:p.Ala5Val	rs121912442	Missense
NM_000454.4:c.20G >T	NP_000445.1:p.Cys7Phe	rs121912448	Missense
NM_000454.4:c.13G >A	NP_000445.1:p.Ala5Thr	rs121912444	Missense
NM_000454.4:c.37G >C	NP_000445.1:p.Gly13Arg	rs121912456	Missense
NM_000454.4:c.49G >A	NP_000445.1:p.Gly17Ser	rs121912453	Missense
NM_000454.4:c.64G >A	NP_000445.1:p.Glu22Lys	rs121912450	Missense
NM_000454.4:c.115C >G	NP_000445.1:p.Leu39Val	rs121912432	Missense
NM_000454.4:c.112G >C	NP_000445.1:p.Gly38Arg	rs121912431	Missense
NM_000454.4:c.112G >A	NP_000445.1:p.Gly38Arg	rs121912431	Missense
NM_000454.4:c.131A >G	NP_000445.1:p.His44Arg	rs121912435	Missense
NM_000454.4:c.125G >A	NP_000445.1:p.Gly42Asp	rs121912434	Missense
NM_000454.4:c.140A >G	NP_000445.1:p.His47Arg	rs121912443	Missense
NM_000454.4:c.124G >A	NP_000445.1:p.Gly42Ser	rs121912433	Missense
NM_000454.4:c.137T >G	NP_000445.1:p.Phe46Cys	rs121912457	Missense
NM_000454.4:c.217G >A	NP_000445.1:p.Gly73Ser	rs121912455	Missense
NM_000454.4:c.242A >G	NP_000445.1:p.His81Arg	rs121912458	Missense
NM_000454.4:c.256G >C	NP_000445.1:p.Gly86Arg	rs121912436	Missense
NM_000454.4:c.253T >G	NP_000445.1:p.Leu85Val	rs121912452	Missense
NM_000454.4:c.281G >C	NP_000445.1:p.Gly94Ala	rs121912438	Missense
NM_000454.4:c.280G >T	NP_000445.1:p.Gly94Cys	rs121912437	Missense
NM_000454.4:c.280G >C	NP_000445.1:p.Gly94Arg	rs121912437	Missense
NM_000454.4:c.289G >A	NP_000445.1:p.Asp97Asn	rs121912459	Missense
NM_000454.4:c.302A >G	NP_000445.1:p.Glu101Gly	rs121912439	Missense
NM_000454.4:c.319C >G	NP_000445.1:p.Leu107Val	rs121912440	Missense
LRG_652t1:c.317C >T	LRG_652p1:p.Ser106Leu		Missense
NM_000454.4:c.313A >T	NP_000445.1:p.Ile105Phe	rs121912445	Missense
NM_000454.4:c.341T >C	NP_000445.1:p.Ile114Thr	rs121912441	Missense
NM_000454.4:c.338T >C	NP_000445.1:p.Ile113Thr	rs74315452	Missense
NM_000454.4:c.358-10T >G			Intron variant
NM_000454.4:c.358-11A >G			Intron variant
NM_000454.4:c.380T >A	NP_000445.1:p.Leu127Ter	rs121912454	Stop gain
NM_000454.4:c.404G >A	NP_000445.1:p.Ser135Asn	rs121912451	Missense
NM_000454.4:c.436G >A	NP_000445.1:p.Ala146Thr	rs121912447	Missense
NM_000454.4:c.435G >C	NP_000445.1:p.Leu145Phe	rs1482760341	Missense
NM_000454.4:c.434T >C	NP_000445.1:p.Leu145Ser	rs121912446	Missense
NM_000454.4:c.455T >C	NP_000445.1:p.Ile152Thr	rs121912449	Missense
**C9orf72**			
NM_001256054.2:c. − 45 + 163GGGGCC[>24]		rs143561967	Intron variant
**FUS**			
NM_004960.3:c.616G >A	NP_004951.1:p.Gly206Ser	rs387906628	Missense
NM_004960.3:c.646C >T	NP_004951.1:p.Arg216Cys	rs267606832	Missense
NM_004960.3:c.1507_1508AG [3]	NP_004951.1:p.Gly504fs		Frameshift
NM_004960.3:c.1483C >T	NP_004951.1:p.Arg495Ter	rs387906627	Stop gain
NM_004960.3:c.1520G >A	NP_004951.1:p.Gly507Asp	rs267606831	Missense
NM_004960.3:c.1570A >T	NP_004951.1:p.Arg524Trp	rs267606833	Missense
NM_004960.3:c.1562G >A	NP_004951.1:p.Arg521His	rs121909671	Missense
NM_004960.3:c.1561C >T	NP_004951.1:p.Arg521Cys	rs121909668	Missense
NM_004960.3:c.1561C >G	NP_004951.1:p.Arg521Gly	rs121909668	Missense
NM_004960.3:c.1553G >A	NP_004951.1:p.Arg518Lys	rs121909669	Missense
NM_004960.3:c.1551C >G	NP_004951.1:p.His517Gln	rs121909667	Missense
TARDBP			
NM_007375.3:c.800A >G	NP_031401.1:p.Asn267Ser	rs80356718	Missense
NM_007375.3:c.869G >C	NP_031401.1:p.Gly290Ala	rs121908395	Missense
NM_007375.3:c.881G >T	NP_031401.1:p.Gly294Val	rs80356721	Missense
NM_007375.3:c.881G >C	NP_031401.1:p.Gly294Ala	rs80356721	Missense
NM_007375.3:c.883G >A	NP_031401.1:p.Gly295Ser	rs80356723	Missense
NM_007375.3:c.892G >A	NP_031401.1:p.Gly298Ser	rs4884357	Missense
NM_007375.3:c.943G >A	NP_031401.1:p.Ala315Thr	rs80356726	Missense
NM_007375.3:c.991C >A	NP_031401.1:p.Gln331Lys	rs80356727	Missense
NM_007375.3:c.1009A >G	NP_031401.1:p.Met337Val	rs80356730	Missense
NM_007375.3:c.1028A >G	NP_031401.1:p.Gln343Arg	rs80356731	Missense
NM_007375.3:c.1042G >T	NP_031401.1:p.Gly348Cys	rs80356733	Missense
NM_007375.3:c.1055A >G	NP_031401.1:p.Asn352Ser	rs80356734	Missense
NM_007375.3:c.1144G >A	NP_031401.1:p.Ala382Thr	rs367543041	Missense
NM_007375.3:c.1153T >G	NP_031401.1:p.Trp385Gly	rs797044595	Missense
